# Supplementary material for: Thousands of Women’s Lives Depend on the Improvement of Poland’s Cervical Cancer Screening and Prevention Education as Well as Better Networking Strategies Amongst Cervical Cancer Facilities
Source: Diagnostics (Basel). 2022 Jul 26;12(8):1807. doi: 10.3390/diagnostics12081807 (PMC9394414; doi:10.3390/diagnostics12081807)
Supplement: Supplementary file 1 [file diagnostics-12-01807-s001.zip › diagnostics-1822651-supplementary.pdf]

Supplementary Material S1

Case of 31-year-old female patient diagnosed with cervical cancer FIGO IIB at the age of 22

As of April 2013, the patient was diagnosed with carcinoma planoepitheliale (G3) akeratodes coli uteri. It was noted during the anamnesis that the patient experienced early sexual intercourse and carried three pregnancies (the first pregnancy was at 17 years old) before the cancer diagnosis. The patient comes from and lives in a small town, she only completed primary school. She also has smoked since the age of 16 years old. Six months before the diagnosis the patient experienced irregular and prolonged menstruation cycles. The patient underwent a surgical treatment with ovarian transposition and pelvic lymphadenectomy, she also had radio-chemotherapy supplemented with brachytherapy. The post-operative complication of an entero-vaginal fistula was treated by implementing a colostomy. From the time of the diagnosis the patient now regularly has visits at the gynecological oncology clinic. As of June 2022, 9 years after the diagnosis, the recurrence of the CC cannot be ruled out. It is necessary to determine the etiology of the lesion found in the uterus (Figure).

In this patient, the CC was discovered at an advanced stage; this provided the evidence that the patient did not see a specialist for a prolonged time period and did not attend regular prophylactic visits. Table 1 shows what health costs for the patient and for the public health care system in Poland could be avoided if the patient had been systemically vaccinated and / or had health education been provided at an early stage of education.

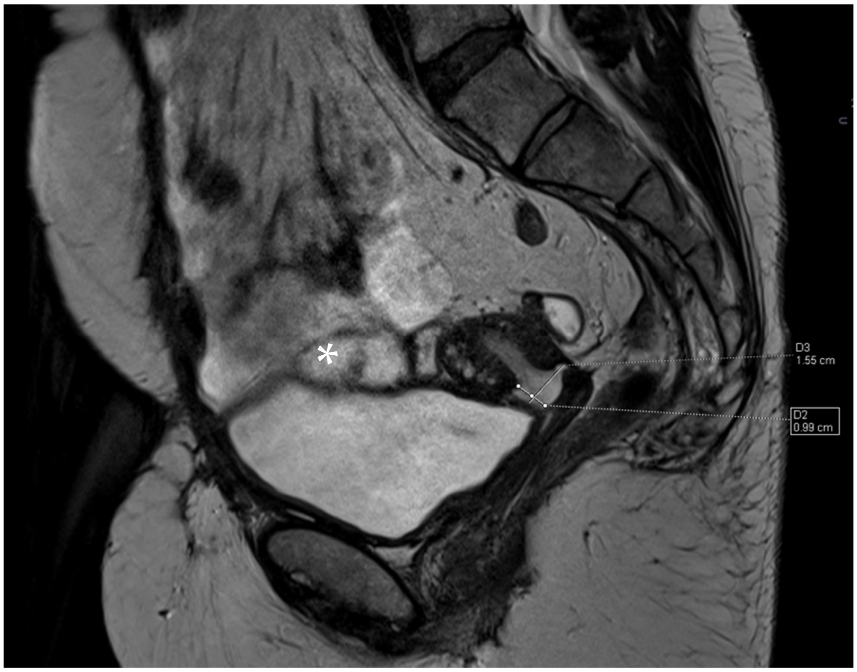

Figure S1. MRI scan of the uterine corpus and cervix - sagittal plane (TSE T2 sequence). *tissue of endometrium-like signal is growing into the scar after the previous cesarean section. The scar is broadened – scar dehiscence features (?). The muscle layer of the lower portion of the anterior uterine wall is significantly reduced. D2, D3 - dimensions of the lesion at the level of the cervix.

Table S1. Avoidable treatment of the patient in the period of 2013–2022.

**Procedures (in order)**
Ovarian transposition, pelvic lymphadenectomy.
Radio-chemotherapy, 5 cycles of DDP.
Brachytherapy, 2 cycles.
FNAB of inguinal nodes.
Inguinal lymphadenectomy.
Removal of entero-vaginal fistula with end-to-end anastomosis.
Reoperation, creating a colostomy.

Legend: DDP – cisplatin; FNAB – fine-needle aspiration biopsy; NHF – National Health Fund
